# *catena*-Poly[[bis­(3,5-dicarboxy­benzo­ato)cobalt(II)]-μ-4,4′-bipyridine]

**DOI:** 10.1107/S1600536808032558

**Published:** 2008-10-15

**Authors:** Chun-Sheng Ling, Qiu-Xia Han

**Affiliations:** a Pharmaceutical College of Henan University, Kaifeng 475004, People’s Republic of China; b Basic Experiment Teaching Center, Henan University, Kaifeng 475004, People’s Republic of China

## Abstract

In the title compound, [Co(C_9_H_5_O_6_)_2_(C_10_H_8_N_2_)]_*n*_, the asymmetric unit consists of one Co^2+^ ion with site symmetry 2, one mono-deprotonated 1,3,5-benzene­tricarboxylic acid anion and one-half of a 4,4′-bipyridine (4,4′-bipy) mol­ecule, in which two N and two C atoms have site symmetry 2. In the crystal structure, the Co^2+^ centre is coordinated by four O atoms from two bidentate carboxyl­ate groups of two anions and two N atoms of two 4,4′-bipy mol­ecules, resulting in infinite chains propagating in [010]. The cobalt coordination is distorted *trans*-CoO_4_N_2_ octa­hedral and inter­chain O—H⋯O hydrogen bonds complete the structure.

## Related literature

For background, see: Feller *et al.* (2007 ▶); Brown *et al.* (2008 ▶).
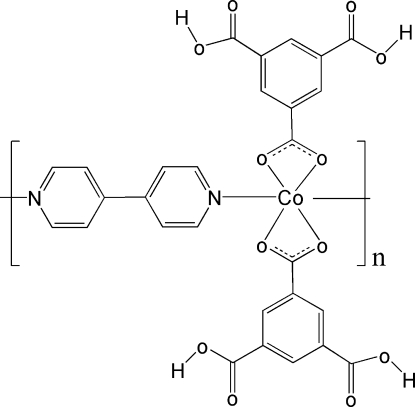


## Experimental

### 

#### Crystal data

[Co(C_9_H_5_O_6_)_2_(C_10_H_8_N_2_)]
*M* *_r_* = 633.37
Monoclinic, 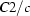

*a* = 10.6682 (7) Å
*b* = 11.0490 (7) Å
*c* = 22.6563 (14) Å
β = 101.401 (1)°
*V* = 2617.9 (3) Å^3^
*Z* = 4
Mo *K*α radiation
μ = 0.73 mm^−1^
*T* = 293 (2) K
0.18 × 0.15 × 0.13 mm

#### Data collection

Bruker SMART CCD diffractometer
Absorption correction: multi-scan (*SADABS*; Sheldrick, 2001 ▶) *T*_min_ = 0.880, *T*_max_ = 0.911
7123 measured reflections
2579 independent reflections
2051 reflections with *I* > 2σ(*I*)
*R*_int_ = 0.031

#### Refinement

*R*[*F* ^2^ > 2σ(*F* ^2^)] = 0.038
*wR*(*F* ^2^) = 0.098
*S* = 1.02
2579 reflections
197 parameters
7 restraints
H-atom parameters constrained
Δρ_max_ = 0.30 e Å^−3^
Δρ_min_ = −0.73 e Å^−3^

### 

Data collection: *SMART* (Bruker, 2001 ▶); cell refinement: *SAINT-Plus* (Bruker, 2001 ▶); data reduction: *SAINT-Plus*); program(s) used to solve structure: *SHELXS97* (Sheldrick, 2008 ▶); program(s) used to refine structure: *SHELXL97* (Sheldrick, 2008 ▶); molecular graphics: *PLATON* (Spek, 2003 ▶); software used to prepare material for publication: *PLATON*.

## Supplementary Material

Crystal structure: contains datablocks global, I. DOI: 10.1107/S1600536808032558/hb2815sup1.cif

Structure factors: contains datablocks I. DOI: 10.1107/S1600536808032558/hb2815Isup2.hkl

Additional supplementary materials:  crystallographic information; 3D view; checkCIF report

## Figures and Tables

**Table 1:** Selected bond lengths (Å)

Co1—N2^i^	1.982 (2)
Co1—N1	1.992 (2)
Co1—O1	2.0221 (14)
Co1—O2	2.4354 (13)

Symmetry code: (i) 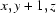.

**Table 2:** Hydrogen-bond geometry (Å, °)

*D*—H⋯*A*	*D*—H	H⋯*A*	*D*⋯*A*	*D*—H⋯*A*
O3—H3⋯O2^ii^	0.81	1.88	2.648 (2)	157
O5—H5⋯O6^iii^	0.78	1.88	2.651 (2)	170

Symmetry codes: (ii) 
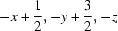
; (iii) 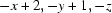.

**Fig. 1.**
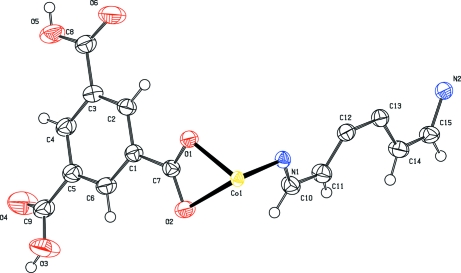
Asymmetric unit of (I), showing displacement ellipsoids at the 50% probability level for the non-hydrogen atoms.

**Fig. 2.**
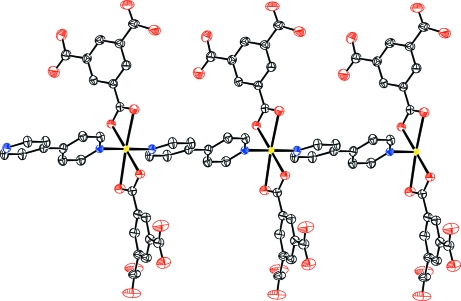
One-dimensional chain structure of (I). H atoms are omitted for clarity. Displacement ellipsoids are drawn at the 50% probability level.

**Fig. 3.**
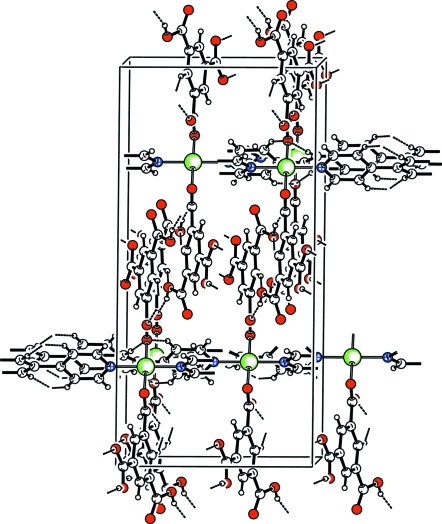
Three-dimensional structure of (I) arising by means of hydrogen bonds. Displacement ellipsoids are drawn at the 50% probability level.

## supplementary crystallographic information

### Comment

Recently, many efforts in coordination chemistry and crystal engineering have been devoted to the construction of metal-organic coordination polymers (MOCPs) employing both coordination bonds and/or hydrogen bonds, due to their appropriate strength and directionality (Feller *et al.* 2007). Dual-ligand or multidentate organic ligands are usually engaged in the construction of MOCPs, among which carboxylates and N,*N*-bidentate ligands are all the simplest connectors potentially able to bridge metal ions (Brown *et al.* 2008). Herein, we report the title compound (I) containing organic dual-ligands (Fig. 1).

The structure of (I) presents a one-dimensional infinite chain (Fig.2), in which the Co^2+^ centre (site symmetry 2) is coordinated by four O atoms from two bidentate carboxylate groups of two 1,3,5-benzenetricarboxylic acid anions, two N atoms of two 4,4'-bipyridine molecules. The Co^2+^ caion resides in a distorted octahedral configuration. In the equatorial plane, it is chelted by four carboxylate oxygen atoms (O1, O2 and their symmetry equivalents) from two 1,3,5-benzenetricarboxylic acid anions (Table 1), in which the Co—O distances are very different.

In addition, these one-dimensional chains are linked together by O—H···O hydrogen bonds between carboxylate groups generating a three-dimensional framework (Fig. 3 and Table 2).

### Experimental

Solid Co(CH_3_COO)_2_.4H_2_O (1 mmol, 0.245 g) was added to an aqueous solution (25 ml) of 1,3,5-benzenetricarboxylic acid (2 mmol, 0.420 g) and 4,4'-bipyridine (1 mmol, 0.156 g). The mixture was refluxed for two hours at 373 K. The solution was filtered, and the filtrate was kept at room temperature. After ten days, purple blocks of (I) were obtained.

### Refinement

The O-bound H atoms were located in difference Fourier maps and refined as riding in their as-found relative positions with *U*_iso_(H) = 1.5*U*_eq_(O).  The C-bound H atoms were geometrically placed (C—H = 0.93Å) and refined as riding, *U*_iso_(*H*) = 1.2*U*_eq_(C).

### Crystal data

**Table:**

[Co(C_9_H_5_O_6_)_2_(C_10_H_8_N_2_)]	*F*(000) = 1292
*M**_r_* = 633.37	*D*_x_ = 1.607 Mg m^−^^3^
Monoclinic, *C*2/*c*	Mo *K*α radiation, λ = 0.71073 Å
Hall symbol: -C 2yc	Cell parameters from 1750 reflections
*a* = 10.6682 (7) Å	θ = 2.7–25.9°
*b* = 11.0490 (7) Å	µ = 0.73 mm^−^^1^
*c* = 22.6563 (14) Å	*T* = 293 K
β = 101.401 (1)°	Block, purple
*V* = 2617.9 (3) Å^3^	0.18 × 0.15 × 0.13 mm
*Z* = 4	

### Data collection

**Table:**

Bruker SMART CCD diffractometer	2579 independent reflections
Radiation source: fine-focus sealed tube	2051 reflections with *I* > 2σ(*I*)
graphite	*R*_int_ = 0.031
ω scans	θ_max_ = 26.0°, θ_min_ = 2.7°
Absorption correction: multi-scan (SADABS; Sheldrick, 2001)	*h* = −13→13
*T*_min_ = 0.880, *T*_max_ = 0.911	*k* = −13→13
7123 measured reflections	*l* = −27→15

### Refinement

**Table:**

Refinement on *F*^2^	Primary atom site location: structure-invariant direct methods
Least-squares matrix: full	Secondary atom site location: difference Fourier map
*R*[*F*^2^ > 2σ(*F*^2^)] = 0.038	Hydrogen site location: difmap and geom
*wR*(*F*^2^) = 0.098	H-atom parameters constrained
*S* = 1.02	*w* = 1/[σ^2^(*F*_o_^2^) + (0.0508*P*)^2^ + 1.9201*P*] where *P* = (*F*_o_^2^ + 2*F*_c_^2^)/3
2579 reflections	(Δ/σ)_max_ = 0.001
197 parameters	Δρ_max_ = 0.31 e Å^−^^3^
7 restraints	Δρ_min_ = −0.73 e Å^−^^3^

### Special details

**Table:**

Geometry. All e.s.d.'s (except the e.s.d. in the dihedral angle between two l.s. planes) are estimated using the full covariance matrix. The cell e.s.d.'s are taken into account individually in the estimation of e.s.d.'s in distances, angles and torsion angles; correlations between e.s.d.'s in cell parameters are only used when they are defined by crystal symmetry. An approximate (isotropic) treatment of cell e.s.d.'s is used for estimating e.s.d.'s involving l.s. planes.
Refinement. Refinement of *F*^2^ against ALL reflections. The weighted *R*-factor *wR* and goodness of fit *S* are based on *F*^2^, conventional *R*-factors *R* are based on *F*, with *F* set to zero for negative *F*^2^. The threshold expression of *F*^2^ > σ(*F*^2^) is used only for calculating *R*-factors(gt) *etc*. and is not relevant to the choice of reflections for refinement. *R*-factors based on *F*^2^ are statistically about twice as large as those based on *F*, and *R*- factors based on ALL data will be even larger.

### Fractional atomic coordinates and isotropic or equivalent isotropic displacement parameters (Å^2^)

**Table:**

	*x*	*y*	*z*	*U*_iso_*/*U*_eq_	
Co1	0.5000	0.64801 (3)	0.2500	0.01788 (9)	
O1	0.60733 (12)	0.64666 (13)	0.18575 (6)	0.0280 (3)	
O2	0.40351 (13)	0.65638 (14)	0.14327 (6)	0.0347 (4)	
O3	0.30786 (17)	0.7752 (2)	−0.06851 (8)	0.0661 (6)	
H3	0.2523	0.7863	−0.0982	0.099*	
O4	0.39825 (18)	0.6736 (2)	−0.13349 (8)	0.0692 (6)	
O5	0.85792 (15)	0.55679 (17)	−0.04477 (7)	0.0509 (5)	
H5	0.9287	0.5390	−0.0439	0.076*	
O6	0.91135 (16)	0.5199 (2)	0.05406 (8)	0.0591 (6)	
N1	0.5000	0.4678 (2)	0.2500	0.0232 (4)	
N2	0.5000	−0.1726 (2)	0.2500	0.0274 (6)	
C1	0.55508 (19)	0.64578 (18)	0.07857 (9)	0.0281 (5)	
C2	0.67509 (19)	0.60572 (19)	0.07160 (9)	0.0298 (5)	
H2	0.7360	0.5843	0.1053	0.036*	
C3	0.70461 (19)	0.5975 (2)	0.01464 (10)	0.0306 (5)	
C4	0.6137 (2)	0.6293 (2)	−0.03605 (10)	0.0338 (5)	
H4	0.6326	0.6222	−0.0742	0.041*	
C5	0.4946 (2)	0.6717 (2)	−0.02928 (10)	0.0329 (5)	
C6	0.4664 (2)	0.6797 (2)	0.02785 (10)	0.0327 (5)	
H6	0.3867	0.7084	0.0322	0.039*	
C7	0.51892 (19)	0.65013 (18)	0.13895 (9)	0.0265 (5)	
C8	0.8332 (2)	0.5551 (2)	0.00808 (10)	0.0362 (6)	
C9	0.3971 (2)	0.7059 (2)	−0.08302 (10)	0.0392 (6)	
C10	0.4034 (2)	0.40519 (19)	0.21766 (10)	0.0321 (5)	
H10	0.3356	0.4477	0.1947	0.039*	
C11	0.3993 (2)	0.28136 (19)	0.21669 (10)	0.0330 (5)	
H11	0.3295	0.2414	0.1938	0.040*	
C12	0.5000	0.2156 (3)	0.2500	0.0284 (7)	
C13	0.5000	0.0812 (3)	0.2500	0.0269 (7)	
C14	0.38822 (19)	0.01470 (19)	0.23157 (10)	0.0333 (5)	
H14	0.3108	0.0547	0.2192	0.040*	
C15	0.3916 (2)	−0.1093 (2)	0.23148 (10)	0.0328 (5)	
H15	0.3158	−0.1514	0.2181	0.039*	

### Atomic displacement parameters (Å^2^)

**Table:**

	*U*^11^	*U*^22^	*U*^33^	*U*^12^	*U*^13^	*U*^23^
Co1	0.02150 (17)	0.01328 (16)	0.01973 (17)	0.000	0.00619 (13)	0.000
O1	0.0246 (6)	0.0345 (8)	0.0250 (7)	0.0022 (6)	0.0052 (5)	0.0008 (6)
O2	0.0252 (7)	0.0480 (9)	0.0314 (8)	0.0040 (7)	0.0071 (6)	0.0026 (7)
O3	0.0446 (10)	0.1163 (17)	0.0354 (10)	0.0378 (11)	0.0033 (8)	0.0035 (10)
O4	0.0557 (11)	0.1214 (18)	0.0293 (10)	0.0270 (12)	0.0052 (8)	−0.0022 (10)
O5	0.0367 (8)	0.0734 (12)	0.0478 (10)	0.0152 (8)	0.0210 (7)	0.0025 (9)
O6	0.0376 (9)	0.0938 (15)	0.0466 (10)	0.0261 (9)	0.0100 (8)	0.0094 (10)
N1	0.0263 (7)	0.0191 (7)	0.0246 (7)	0.000	0.0062 (6)	0.000
N2	0.0268 (12)	0.0246 (13)	0.0313 (13)	0.000	0.0072 (10)	0.000
C1	0.0268 (10)	0.0300 (10)	0.0276 (10)	0.0007 (9)	0.0057 (8)	−0.0005 (9)
C2	0.0250 (10)	0.0327 (11)	0.0308 (11)	0.0034 (9)	0.0030 (9)	0.0013 (9)
C3	0.0254 (10)	0.0333 (11)	0.0335 (11)	0.0015 (9)	0.0070 (9)	−0.0025 (9)
C4	0.0294 (10)	0.0441 (13)	0.0296 (11)	0.0011 (10)	0.0097 (9)	−0.0015 (10)
C5	0.0289 (11)	0.0410 (13)	0.0288 (11)	0.0011 (9)	0.0054 (9)	0.0003 (9)
C6	0.0256 (10)	0.0413 (12)	0.0320 (12)	0.0031 (9)	0.0071 (9)	−0.0010 (10)
C7	0.0259 (10)	0.0244 (10)	0.0291 (10)	0.0014 (8)	0.0050 (8)	0.0002 (9)
C8	0.0297 (11)	0.0435 (13)	0.0369 (13)	0.0022 (10)	0.0100 (10)	−0.0008 (10)
C9	0.0292 (11)	0.0614 (15)	0.0284 (12)	0.0044 (11)	0.0092 (9)	0.0027 (11)
C10	0.0317 (11)	0.0265 (11)	0.0370 (12)	0.0027 (9)	0.0042 (9)	0.0031 (9)
C11	0.0310 (11)	0.0271 (11)	0.0391 (12)	−0.0005 (9)	0.0030 (9)	−0.0002 (9)
C12	0.0267 (14)	0.0246 (15)	0.0354 (16)	0.000	0.0096 (12)	0.000
C13	0.0270 (14)	0.0238 (15)	0.0300 (16)	0.000	0.0057 (12)	0.000
C14	0.0246 (10)	0.0265 (11)	0.0464 (13)	0.0007 (9)	0.0015 (10)	0.0022 (10)
C15	0.0241 (10)	0.0276 (11)	0.0456 (13)	−0.0015 (9)	0.0044 (10)	0.0006 (10)

### Geometric parameters (Å, °)

**Table:**

Co1—N2^i^	1.982 (2)	C1—C7	1.494 (3)
Co1—N1	1.992 (2)	C2—C3	1.390 (3)
Co1—O1^ii^	2.0221 (14)	C2—H2	0.9300
Co1—O1	2.0221 (14)	C3—C4	1.393 (3)
Co1—O2^ii^	2.4354 (13)	C3—C8	1.485 (3)
Co1—O2	2.4354 (13)	C4—C5	1.391 (3)
O1—C7	1.273 (2)	C4—H4	0.9300
O2—C7	1.256 (2)	C5—C6	1.389 (3)
O3—C9	1.314 (3)	C5—C9	1.485 (3)
O3—H3	0.8127	C6—H6	0.9300
O4—C9	1.200 (3)	C10—C11	1.369 (3)
O5—C8	1.276 (3)	C10—H10	0.9300
O5—H5	0.7761	C11—C12	1.390 (3)
O6—C8	1.260 (3)	C11—H11	0.9300
N1—C10	1.333 (2)	C12—C11^ii^	1.390 (3)
N1—C10^ii^	1.333 (2)	C12—C13	1.485 (4)
N2—C15^ii^	1.346 (2)	C13—C14	1.392 (2)
N2—C15	1.346 (2)	C13—C14^ii^	1.392 (2)
N2—Co1^iii^	1.982 (2)	C14—C15	1.371 (3)
C1—C6	1.388 (3)	C14—H14	0.9300
C1—C2	1.393 (3)	C15—H15	0.9300
			
N2^i^—Co1—N1	180.0	C1—C6—C5	121.1 (2)
N2^i^—Co1—O1^ii^	90.42 (4)	C1—C6—H6	119.5
N1—Co1—O1^ii^	89.58 (4)	C5—C6—H6	119.5
N2^i^—Co1—O1	90.42 (4)	O2—C7—O1	120.87 (19)
N1—Co1—O1	89.58 (4)	O2—C7—C1	120.51 (18)
O1^ii^—Co1—O1	179.16 (8)	O1—C7—C1	118.62 (17)
C7—O1—Co1	99.65 (12)	O6—C8—O5	123.7 (2)
C9—O3—H3	109.1	O6—C8—C3	119.2 (2)
C8—O5—H5	110.6	O5—C8—C3	117.11 (19)
C10—N1—C10^ii^	117.5 (2)	O4—C9—O3	123.8 (2)
C10—N1—Co1	121.23 (12)	O4—C9—C5	124.6 (2)
C10^ii^—N1—Co1	121.23 (12)	O3—C9—C5	111.60 (19)
C15^ii^—N2—C15	117.4 (2)	N1—C10—C11	123.1 (2)
C15^ii^—N2—Co1^iii^	121.30 (12)	N1—C10—H10	118.5
C15—N2—Co1^iii^	121.30 (12)	C11—C10—H10	118.5
C6—C1—C2	118.9 (2)	C10—C11—C12	119.7 (2)
C6—C1—C7	119.49 (18)	C10—C11—H11	120.2
C2—C1—C7	121.58 (18)	C12—C11—H11	120.2
C3—C2—C1	120.52 (19)	C11^ii^—C12—C11	116.9 (3)
C3—C2—H2	119.7	C11^ii^—C12—C13	121.54 (13)
C1—C2—H2	119.7	C11—C12—C13	121.54 (13)
C2—C3—C4	120.00 (19)	C14—C13—C14^ii^	116.3 (3)
C2—C3—C8	119.79 (18)	C14—C13—C12	121.83 (13)
C4—C3—C8	120.2 (2)	C14^ii^—C13—C12	121.83 (13)
C5—C4—C3	119.7 (2)	C15—C14—C13	120.4 (2)
C5—C4—H4	120.1	C15—C14—H14	119.8
C3—C4—H4	120.1	C13—C14—H14	119.8
C6—C5—C4	119.72 (19)	N2—C15—C14	122.7 (2)
C6—C5—C9	120.1 (2)	N2—C15—H15	118.7
C4—C5—C9	120.2 (2)	C14—C15—H15	118.7
			
N2^i^—Co1—O1—C7	−88.18 (11)	C6—C1—C7—O1	−163.76 (19)
N1—Co1—O1—C7	91.82 (11)	C2—C1—C7—O1	17.7 (3)
O1^ii^—Co1—O1—C7	91.82 (12)	C2—C3—C8—O6	4.3 (3)
N2^i^—Co1—N1—C10	−141 (22)	C4—C3—C8—O6	−175.8 (2)
O1^ii^—Co1—N1—C10	83.93 (12)	C2—C3—C8—O5	−175.6 (2)
O1—Co1—N1—C10	−96.07 (12)	C4—C3—C8—O5	4.3 (3)
N2^i^—Co1—N1—C10^ii^	39 (23)	C6—C5—C9—O4	−159.5 (3)
O1^ii^—Co1—N1—C10^ii^	−96.07 (12)	C4—C5—C9—O4	19.2 (4)
O1—Co1—N1—C10^ii^	83.93 (12)	C6—C5—C9—O3	19.8 (3)
C6—C1—C2—C3	−1.3 (3)	C4—C5—C9—O3	−161.5 (2)
C7—C1—C2—C3	177.20 (19)	C10^ii^—N1—C10—C11	0.39 (16)
C1—C2—C3—C4	−0.1 (3)	Co1—N1—C10—C11	−179.61 (16)
C1—C2—C3—C8	179.8 (2)	N1—C10—C11—C12	−0.8 (3)
C2—C3—C4—C5	1.4 (3)	C10—C11—C12—C11^ii^	0.37 (15)
C8—C3—C4—C5	−178.4 (2)	C10—C11—C12—C13	−179.63 (15)
C3—C4—C5—C6	−1.3 (3)	C11^ii^—C12—C13—C14	161.59 (15)
C3—C4—C5—C9	180.0 (2)	C11—C12—C13—C14	−18.41 (15)
C2—C1—C6—C5	1.5 (3)	C11^ii^—C12—C13—C14^ii^	−18.41 (15)
C7—C1—C6—C5	−177.08 (19)	C11—C12—C13—C14^ii^	161.59 (15)
C4—C5—C6—C1	−0.2 (3)	C14^ii^—C13—C14—C15	−0.70 (16)
C9—C5—C6—C1	178.6 (2)	C12—C13—C14—C15	179.30 (16)
Co1—O1—C7—O2	1.9 (2)	C15^ii^—N2—C15—C14	−0.74 (16)
Co1—O1—C7—C1	−177.68 (15)	Co1^iii^—N2—C15—C14	179.26 (16)
C6—C1—C7—O2	16.6 (3)	C13—C14—C15—N2	1.5 (3)
C2—C1—C7—O2	−161.9 (2)		

Symmetry codes: (i) *x*, *y*+1, *z*; (ii) −*x*+1, *y*, −*z*+1/2; (iii) *x*, *y*−1, *z*.

### Hydrogen-bond geometry (Å, °)

**Table:**

*D*—H···*A*	*D*—H	H···*A*	*D*···*A*	*D*—H···*A*
O3—H3···O2^iv^	0.81	1.88	2.648 (2)	157
O5—H5···O6^v^	0.78	1.88	2.651 (2)	170

Symmetry codes: (iv) −*x*+1/2, −*y*+3/2, −*z*; (v) −*x*+2, −*y*+1, −*z*.
